# Weight-bearing CT as an approach to assess femoral–acetabular displacement during external rotation stress in the hip

**DOI:** 10.1093/jhps/hnaf001

**Published:** 2025-01-22

**Authors:** Dominic J. L Rivas, Joshua M Gassmann, Jessica E Goetz, Holly D Aitken, John C Davison, Aspen Miller, Michael C Willey

**Affiliations:** University of Iowa Hospitals and Clinics, Department of Orthopedics and Rehabilitation, 200 Hawkins Dr., Iowa City, IA 52242, United States; University of Iowa Hospitals and Clinics, Department of Orthopedics and Rehabilitation, 200 Hawkins Dr., Iowa City, IA 52242, United States; University of Iowa Hospitals and Clinics, Department of Orthopedics and Rehabilitation, 200 Hawkins Dr., Iowa City, IA 52242, United States; University of Iowa Hospitals and Clinics, Department of Orthopedics and Rehabilitation, 200 Hawkins Dr., Iowa City, IA 52242, United States; University of Iowa Hospitals and Clinics, Department of Orthopedics and Rehabilitation, 200 Hawkins Dr., Iowa City, IA 52242, United States; University of Iowa Hospitals and Clinics, Department of Orthopedics and Rehabilitation, 200 Hawkins Dr., Iowa City, IA 52242, United States; University of Iowa Hospitals and Clinics, Department of Orthopedics and Rehabilitation, 200 Hawkins Dr., Iowa City, IA 52242, United States

## Abstract

Hip dysplasia causes pathologic joint mechanics and can produce hip instability, leading to progressive joint degeneration and osteoarthritis. Weight-bearing computed tomography (WBCT) is an emerging technology that may enable quantification of femoral–acetabular displacement as an objective indicator of instability. To evaluate this potential, 10 patients indicated for periacetabular osteotomy to treat hip dysplasia and 10 healthy controls underwent two WBCT protocols. Participants were scanned in a neutral stance [weight-bearing (WB)] and again with the hip stressed in maximal external rotation (WB-stress), a position hypothesized to reproduce anterior instability. Clinical, nonweight-bearing computed tomography (CT) scans were available for patients with hip dysplasia. Congruency of the femoroacetabular joint space and position of the femoral head in the acetabulum were quantified via multiple 2D manual measurements and automated 3D measurements. There were no 2D measurements found to differ between the WB and WB-stress scans in either dysplastic (*P *= .742–1.000) or control (*P *= .203–1.000) hips. 3D translation of the femoral head center from WB to WB-stress averaged 1.3 ± 0.6 mm in the control hips, compared to 0.9 ± 0.4 mm in the dysplastic hips (*P *= .096). 3D joint space width (JSW) was determined for both the control and dysplastic hips, with greater JSW found in control hips for both the WB (*P *= .049) and WB-stress (*P *= .003) scans. WBCT has the potential to better capture subtle femoral–acetabular displacement derived from both automated 3D and manual 2D measurements in static instability-prone joint orientations.

## Introduction

Hip dysplasia is a musculoskeletal deformity of the proximal femur and acetabulum that results in a variable combination of joint instability and pathologic loading that leads to the development of osteoarthritis [1]. Periacetabular osteotomy (PAO) is the gold standard surgical treatment for prearthritic hip dysplasia in adolescents and young adults with a lateral center edge angle of Wiberg (LCEA) less than 20°. Satisfactory clinical outcomes can be obtained in this population greater than 10 years after surgery [2–6]. However, there is significant controversy surrounding the surgical treatment of borderline hip dysplasia (LCEA 20°–25° or 18°–25°). Many surgeons report good short-term outcomes of borderline hip dysplasia treated with hip arthroscopy alone [7–10], but there are also reports of persistent/recurrent dysfunction/instability requiring revision surgery with PAO [11–16]. Additionally, excellent outcomes can be achieved with select borderline dysplastic hips with PAO [17].

Hips with borderline dysplasia and features of instability (Tönnis angle >10°; anterior center edge angle <20°; alpha angle <55°; internal rotation in flexion >20°) treated with PAO have excellent outcomes [17], but borderline dysplastic hips with femoroacetabular impingement and lack of perceived instability (anterior groin pain with abduction, extension, and external rotation, or excessive external rotation range of motion) may be treated successfully with hip arthroscopy [9]. An objective, quantitative assessment of functional joint stability may be a useful tool to help guide appropriate surgical treatment. However, beyond subjective clinical exam [18–23], there are limited tools and techniques that allow clinicians to objectively quantify hip joint instability, and no clear definition of what amount of femoral–acetabular displacement is indicative of instability.

Standing weight-bearing CT (WBCT) is an emerging technology that permits clinical evaluation of lower extremity bony alignment while an individual is in a loaded, functional position [24–27]. WBCT employs comparable radiation doses in a faster acquisition time compared to clinical X-ray and lower doses than standard supine CT [28–32], which makes it a potentially useful imaging modality for detailed evaluation of bone orientation in multiple positions. The use of this technology to visualize asymmetric distances between the femoral head and the acetabulum during loading, and to provide an objective assessment of displacement within the joint, could inform the establishment and objective diagnosis of hip instability. Therefore, the aims of this study were to (1) establish reliable 2D and 3D measurement techniques to assess femoral head displacement in the acetabulum on hip scans, (2) compare measurements made on clinical nonweight-bearing (NWB) CT and standing WBCT scans, and (3) determine if WBCT can detect differences in femoral–acetabular displacement between dysplastic hips and hips without dysplasia. We hypothesized that a maximal external rotation stress position, similar to that used in diagnostic physical examinations to reproduce anterior instability, within the WBCT would elicit detectable femoral head center translation relative to the acetabulum, and that femoral head center translation would be greater within the acetabulum of dysplastic hips than nondysplastic hips.

## Materials and methods

**Figure 1.**
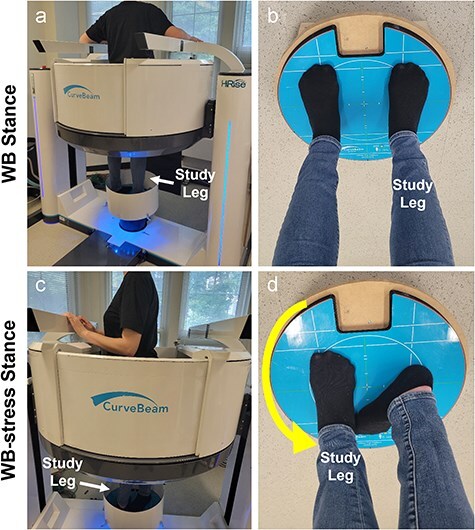
(a) Participant standing in the Curvebeam High Rise® weight-bearing CT scanner for the neutral WBCT scan of the hip. (b) Demonstration of the foot position on the foot platform for the neutral (WB) scan. (c) Patient inside the WBCT scanner demonstrating patient-selected maximal external rotation during the stress (WB-stress) scan. (d) The foot on the study leg was shifted to the foot location of the contralateral leg during the neutral scan, and the patient was instructed to rotate their body on the study leg to bring their opposite shoulder as far backward as possible. For this case, the right hip is the study hip, so the left shoulder was rotated back in the scanner (toward the camera/viewer). The participant then brought their contralateral foot back onto the platform behind the foot of the study limb (d) and equalized weight for stability during scanning.

With institutional review board approval, we enrolled 10 individuals (9 females) with hip dysplasia indicated for PAO [27] and 10 control subjects (8 females) with no reported history of hip pain. Each participant consented in writing to undergo two WBCT scans (HiRise; Curvebeam, Hatfield, PA; 0.3–0.5 mm isotropic voxels). The first was acquired in a relaxed, two-legged standing position with the toes pointed forward on the scanning platform, and the second was acquired with the study hip stressed in maximum external rotation (Fig. 1).

In addition to these study-specific scans, the clinical (diagnostic) NWB CT scan (0.6–0.7 mm isotropic voxel size) and the preoperative standing anteroposterior (AP) pelvis radiographs were extracted from each dysplasia patient’s medical record. LCEA was measured on preoperative standing AP pelvis radiographs of the dysplasia patients [33] and on digitally reconstructed radiographs of control participants [34].

### Manual 2D CT measurements

**Figure 2.**
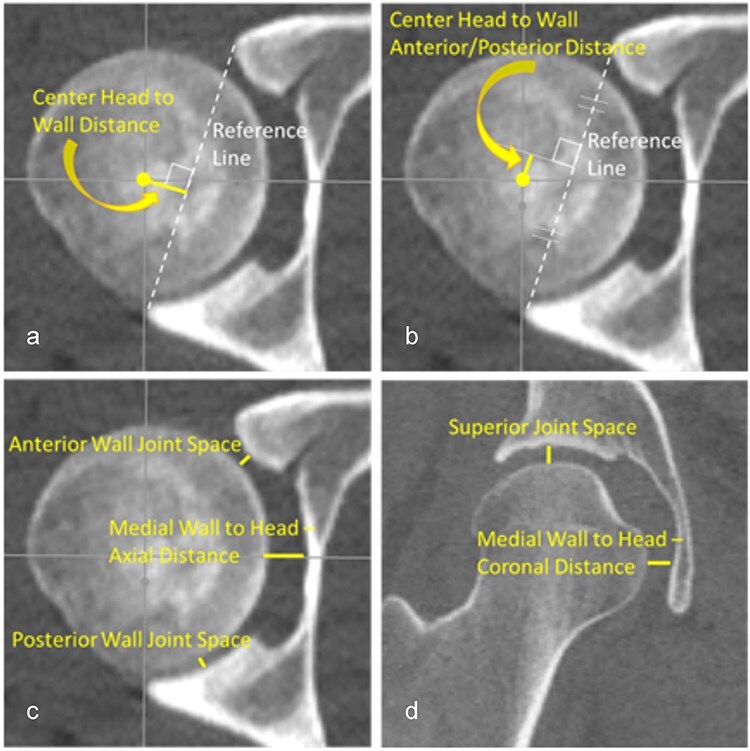
A total of seven 2D manual measurements were performed on each CT scan: (a) the distance from the FHC to a line connecting the anterior and posterior rim of the acetabulum, (b) the distance from the FHC to the perpendicular bisector of the rim-to-rim line, (c) the anterior and posterior wall joint space along with the medial wall to head distance in the axial plane, and (d) the superior joint space and the medial wall to head distance in the coronal plane. All measurements were taken on a single axial and a coronal plane image that was verified in three orthogonal imaging planes to pass through the center of the left and right femoral heads.

2D measurements to evaluate femoral head position in the acetabulum were developed based on similar metrics used to assess fibula position within the incisura of the distal tibia at the ankle (syndesmosis) [24]. Measurements were performed on single axial and coronal CT slices through the left and right femoral head centers (FHCs) by the same investigator (J.M.G.) using RadiAnt® DICOM Viewer (v.2020.2.3). Hip measurements made in the axial plane (Fig. 2) included distance from the FHC to a line connecting the anterior and posterior rim of the acetabulum; distance from the FHC to the perpendicular bisector of the rim-to-rim line; anterior and posterior joint space width; and the medial wall to head distance. In the coronal plane, the superior joint space and the medial wall to femoral head distance were measured (Fig. 2).

### 3D computational measures—geometry

3D surface models of femoral and pelvic geometry were created by segmenting bony contours of each patient’s stressed, unstressed, and NWB CT scans in Mimics (Materalise NV, Leuven, Belgium). To minimize the effect of segmentation differences on computed 3D measures, copies of the bone surfaces least affected by motion artifact (those segmented from the clinical CT scan for dysplastic hips and from the unstressed WBCT segmentations for the control hips) were spatially registered to the associated pelvis and femur surfaces on the stressed and unstressed WBCT using an iterative closest point algorithm (GeoMagic Design X; Systems, Inc., Rock Hill, SC). The amount of axial-plane hip rotation performed by each study participant during the external rotation stress maneuver was determined by decomposing the spatial transformation matrices needed to align the neutral-standing femur and pelvis surface models to their corresponding stressed-standing surfaces into hip rotation angles. The difference between these pelvis and femur angles was the external rotation performed [27].

### 3D computational measures—femoral head translation and joint space width mapping

**Figure 3.**
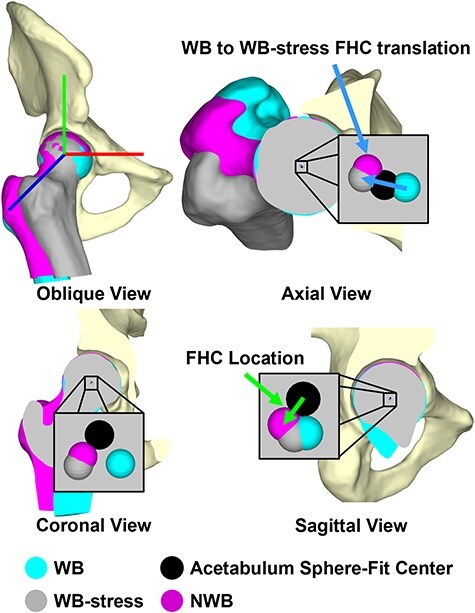
3D Femoral head center location was quantified as the difference in position of the center of a sphere-fit to the articular portion of the femoral head (WB: cyan; NWB: magenta, WB-stress: gray) relative to the center of a sphere-fit to the acetabular lunate (black), while femoral head center translation was calculated as the difference in femoral head center locations [i.e. the external rotation maneuver from WB (cyan) to WB-stress (gray)].

To quantify femoral–acetabular displacement in 3D, translation of the FHC within the acetabulum and changes in joint space width (JSW) were automatically calculated for each scan condition (NWB, WB, WB-stress). To facilitate these calculations, the femoral head and acetabular lunate subchondral bone surfaces were manually isolated from the most accurate segmented pelvis and femur surfaces (dysplasia—NWB scan; control—WB scan). FHC location was defined as the position of the center of a sphere-fit to the articular surface of the femoral head relative to a sphere-fit center of the acetabular lunate in a hip coordinate system [35]. FHC location was defined for each scan condition, and then FHC translation during the external rotation maneuver was calculated as the vector from the WB FHC to the WB-stress FHC location (Fig. 3).

**Figure 4.**
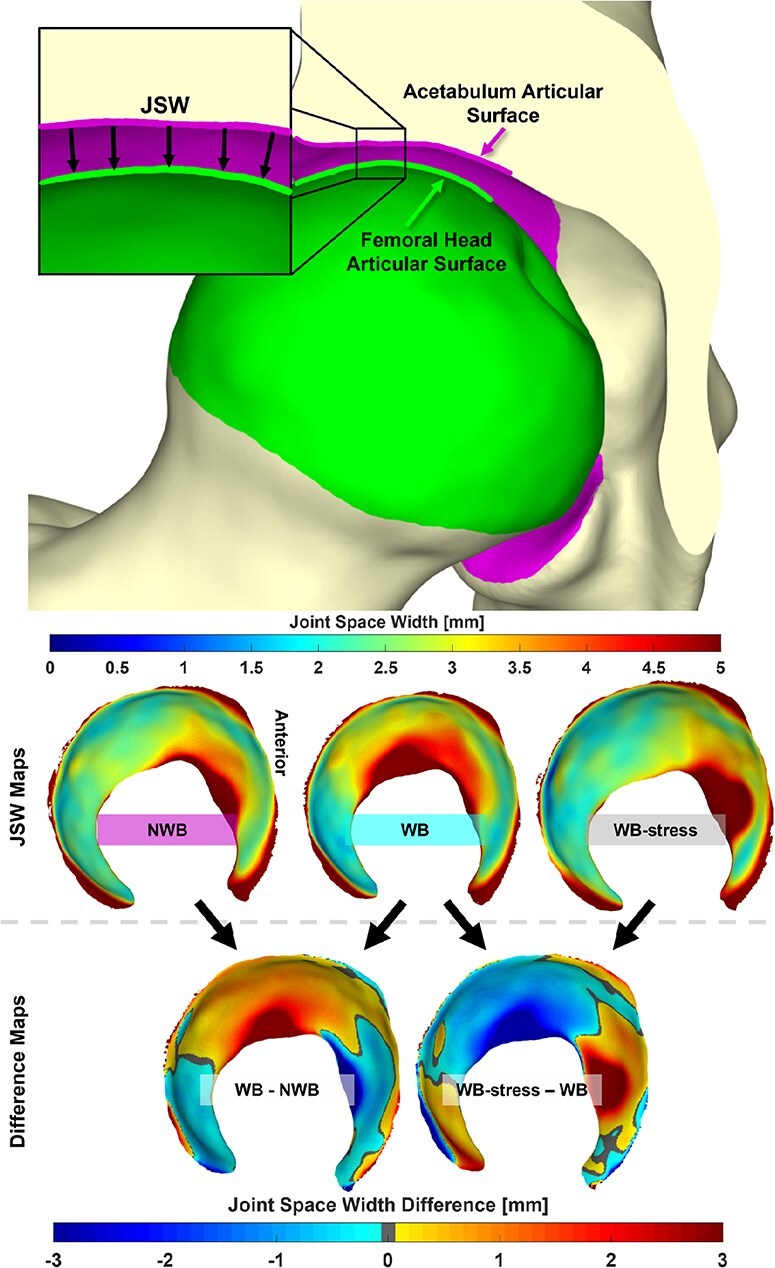
(Top) 3D JSW was defined as the projected distance along the normal of each triangular facet comprising the acetabular articular surface toward the femoral head articular surface. (Middle) JSW was calculated for the NWB, WB, and WB-stress positioned geometries. (Bottom) Comparisons of 3D joint space were made between the WB and both the NWB and WB-stress, with difference maps generated for each patient. Anterior is depicted on the right of all joint space maps.

3D JSW was calculated using a ray-cast method [36]. This process projected a ray normal to each triangular facet comprising the surface of the acetabular lunate toward the femoral head, and the distance traversed by the ray before it intersected the femoral head surface represented the JSW at that location (Fig. 4). JSW was averaged across the entire lunate surface, and also averaged in six separate acetabular subregions to identify changes in JSW indicative of the femoral head shifting within the acetabulum. This subdivision was achieved by defining a plane orthogonal to a line connecting the anterior and posterior inferior acetabular edges and passing through the center of the sphere-fit to the acetabular lunate surface. This plane was then rotated ±45° about a mediolateral axis through the acetabular sphere center to separate posterior, superior, and anterior acetabular subregions, each of which was further subdivided into lateral and medial regions based on half the distance between the medial and lateral edges of the acetabulum [27, 36].

### Statistical analysis

Inter-rater reliability of the 2D manual measurements was determined by calculating the intra-class correlation coefficient (ICC) for measurements from a subset of four dysplastic and four control unstressed WB scans measured by two additional investigators (M.C.W. and J.C.D. along with J.M.G. measurements). Individual 2D measurements and automated 3D measurements were compared between WB, WB-stress, and NWB views for dysplastic participants, and between the WB and WB-stress views for the control group using Wilcoxon signed-rank tests. Wilcoxon rank-sum tests were used to compare the WB and WB-stress measurements between the dysplastic and control groups. Results are presented as median (interquartile range) and in units of millimeters (mm).

Regional analyses of changes in JSW between NWB—WB (dysplasia only), WB—WB-stress, and NWB—WB-stress (dysplasia only) were performed for each of the acetabular lunate subregions. Data were pooled between normal and dysplastic hip types, and Spearman correlations were used to describe the relationships between LCEA and the 2D manual and 3D automated CT measurements. Statistical analyses were performed using SAS software version 9.4 (SAS Institute, Inc., Cary, NC), and for each statistical comparison a Holm–Bonferroni multiple-comparisons correction was performed. A *P*-value ≤ .05 was considered statistically significant.

## Results

Dysplastic patients were 19.6 ± 5.1 years old, had a BMI of 22.9 ± 2.3, and a LCEA of 19.9° ± 2.3°. Controls were 24.2 ± 3.3 years old, had a BMI of 22.3 ± 2.4, and a LCEA of 27.9° ± 5.4°. Patient age (*P *= .041) and LCEA (*P *= .002) were significantly greater in controls compared to dysplastic patients, while BMI (*P *= .418) was not. Axial hip rotation from the unstressed to stressed position in the WBCT averaged 43°± 12° for dysplasia patients and 34°± 17° for control participants (*P *= .256). There was no correlation between LCEA and axial rotation achieved for either the dysplastic patients (rho = -0.018, *P *= .960) or the controls (rho = −0.042, *P *= .907).

### 2D CT-based measurements

Inter-rater reliability (Table 1) ranged from moderate for the “center head to wall” distance (ICC = 0.725; 95% CI: [0.369–0.929]) to poor for the axial-plane “medial wall to head” distance (ICC = −0.177; 95% CI: [−0.409–0.358]). Despite an obvious difference in patient scan orientation, no significant differences were found in any 2D measurement between the NWB and WB (*P *= 1.000) measurements for dysplastic hips. There were also no significant differences in any 2D CT measurements between the WB and WB-stress scans in the dysplastic (*P *= .742–1.000) or the control (*P *= .203–1.000) hips (Table 1). Smaller “posterior joint space” and “center head to wall line-perpendicular distance” were associated with higher LCEA for both the stressed (rho = −0.65, *P *= .002; rho = −0.55, *P *= .012) and unstressed (rho = −0.47, *P *= .002; rho = −0.56, *P *< .001) orientations.

**Table 1.** 2D CT-based measurements of femoral head location in the acetabulum for dysplastic and control hips with WB, WB-stress, and NWB (dysplastic only) orientations Signed rank tests compared scan conditions, while rank-sum tests compared control to dysplastic hips during the same scan conditions. Inter-rater correlation coefficients are shown in column 1. Bold values indicate statistically significant differences (*P* < .05).

		CT scan measure (Median [interquartile range]) [mm]			In-group		Between group	
2D manual CT-based measurements		WB	WB-stress	NWB	WB vs WB-stress	WB vs NWB	WB	WB-stress
Center head to wall distance	Dysplastic	7.6 [6.5–9.0]	8.8 [8.2–9.5]	7.7 [6.7–9.1]	0.742	1.000	0.492	0.270
ICC = 0.725	Control	5.4 [4.9–8.2]	5.5 [5.3–7.4]		1.000			
Center head to wall distance anterior/posterior	Dysplastic	0.0 [−0.5–0.3]	−0.6 [−1.3–0.6]	0.2 [−0.4–0.6]	1.000	1.000	1.000	1.000
ICC = 0.106	Control	−0.4 [−0.8–0.2]	−0.6 [−1.1–0.3]		1.000			
Anterior joint space	Dysplastic	2.9 [2.7–3.4]	3.2 [2.9–3.3]	3.1 [2.9–3.5]	1.000	1.000	0.091	1.000
ICC = 0.473	Control	2.3 [2.2–2.7]	2.9 [2.6–3.4]		0.834			
Posterior joint space	Dysplastic	2.2 [1.7–2.7]	2.2 [1.9–2.7]	2.0 [1.9–2.3]	1.000	1.000	1.000	**0.007**
ICC = 0.099	Control	1.8 [1.6–2.2]	1.5 [1.2–1.8]		0.203			
Medial wall to head - axial distance	Dysplastic	6.6 [5.9–7.0]	6.3 [5.8–7.0]	6.9 [6.1–7.4]	1.000	1.000	1.000	1.000
ICC = 0.177	Control	6.0 [5.8–6.7]	5.8 [5.3–6.8]		1.000			
Superior joint space	Dysplastic	3.8 [3.5–3.9]	3.7 [3.5–4.2]	3.9 [3.2–4.4]	1.000	1.000	1.000	1.000
ICC = 0.452	Control	3.5 [3.2–3.8]	4.4 [3.3–4.5]		1.000			
Medial wall to head - coronal distance	Dysplastic	6.4 [6.2–6.7]	6.4 [6.1–7.0]	6.6 [6.1–7.1]	1.000	1.000	1.000	0.295
ICC = 0.002	Control	6.1 [5.4–6.9]	5.7 [5.1–6.3]		1.000			

### Computed 3D measurements of femoral head movement

**Figure 5.**
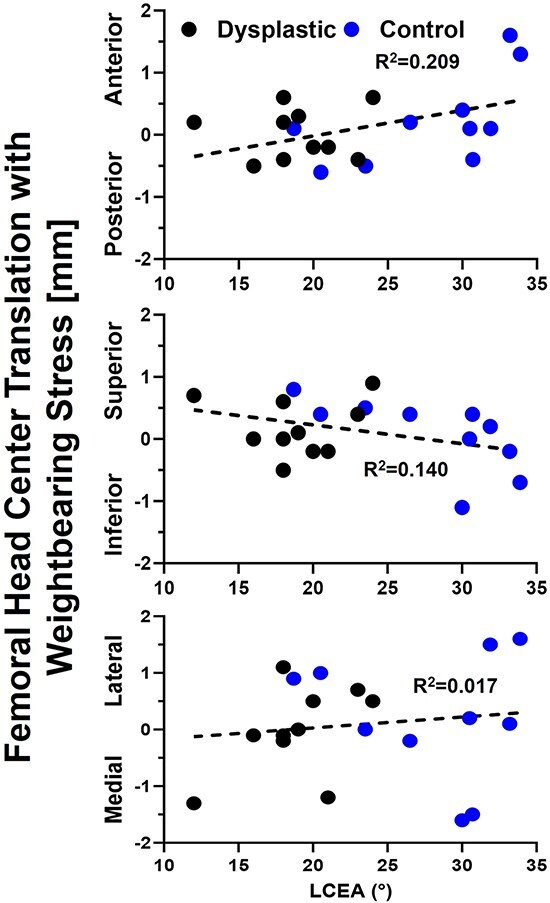
Axis-specific femoral head center translation from the neutral to stressed weight-bearing stance was unrelated to lateral coverage with control hip data shown in blue, dysplastic hip data in black, and the linear regression equation relating translation to lateral coverage as the dashed black line.

Total 3D translation of the FHC between the WB and WB-stress conditions ranged from 0.3 to 1.5 mm (mean: 0.9 mm) for the dysplastic hips. 3D FHC translation for the control hips ranged between 0.2 and 2.2 mm (mean: 1.3). The largest contributor to 3D translation was superior–inferior movement (Fig. 5). Interestingly, in dysplasia patients, the femoral head center location was significantly more posterior (*P *= .042) in the NWB compared to the WBCT, presumably from gravity pulling posteriorly in the NWB scan rather than inferiorly in the WB scans. The location of the femoral head center was not significantly different between the WB and WB-stress conditions within or between hip types (Table 2). There was no relationship between acetabular coverage (LCEA) and FHC translation (rho = 0.40, *P* = .082).

**Table 2.** 3D automated measurements of femoral head center location and JSW for dysplastic and control hips for WB, WB-stress, and NWB (dysplastic only) orientations Bold values indicate statistically significant differences (*P* < 0.05).

			CT scan Average (Standard Deviation) [mm]			In-group		Between group	
3D automated measurements			WB	WB-stress	NWB	WB vs WB-stress	WB vs NWB	WB	WB-stress
Femoral head center location	Anterior/Posterior (+) (−)	Dysplastic	−0.02 (0.31)	0.00 (0.42)	−0.20 (0.22)	1.000	**0.042**	0.650	0.514
		Control	0.11 (0.55)	0.33 (0.73)		1.000			
	Medial/Lateral (−) (+)	Dysplastic	0.03 (0.87)	0.01 (0.88)	0.15 (0.65)	1.000	0.320	0.393	0.246
		Control	0.42 (0.69)	0.62 (0.76)		1.000			
	Superior/Inferior (+) (−)	Dysplastic	−0.62 (0.37)	−0.45 (0.54)	−0.53 (0.47)	0.825	0.322	0.454	0.545
		Control	−0.34 (0.51)	−0.27 (0.67)		1.000			
JSW	Whole-Joint	Dysplastic	3.43 (0.49)	3.20 (0.39)	3.30 (0.54)	0.065	0.065	**0.049**	**0.003**
		Control	3.99 (0.53)	3.90 (0.48)		0.710			
	Anterior lateral	Dysplastic	3.45 (0.67)	4.09 (0.81)	3.62 (0.79)	**0.020**	0.492	0.290	0.427
		Control	3.73 (0.76)	4.35 (0.58)		0.641			
	Superior lateral	Dysplastic	3.43 (0.62)	3.11 (0.74)	3.22 (0.68)	0.090	0.137	**0.008**	**0.016**
		Control	4.58 (0.97)	4.04 (0.65)		0.420			
	Posterior lateral	Dysplastic	3.93 (1.66)	3.16 (1.00)	3.83 (1.68)	0.131	0.418	0.199	0.112
		Control	4.28 (1.26)	4.08 (1.47)		0.375			
	Anterior medial	Dysplastic	3.08 (0.44)	3.47 (0.68)	3.22 (0.65)	0.055	0.262	0.241	0.257
		Control	3.52 (0.75)	3.78 (0.53)		**0.033**			
	Superior medial	Dysplastic	3.64 (0.79)	2.90 (0.58)	3.19 (0.79)	**0.027**	**0.041**	0.571	**0.016**
		Control	3.77 (0.72)	3.71 (0.66)		0.770			
	Posterior medial	Dysplastic	2.88 (0.55)	2.67 (0.56)	2.69 (0.31)	0.361	0.059	0.070	**0.003**
		Control	3.61 (0.92)	3.50 (0.75)		0.846			

**Figure 6.**
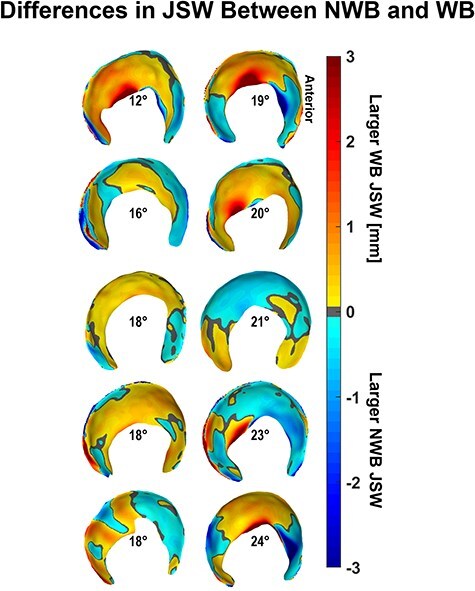
Dysplastic NWB JSW was larger in the anterior subregion and smaller in the medial regions compared to the weight-bearing stance JSW when observed individually rather than collectively, with hips arranged by increasing lateral coverage according to the LCEA shown at the center of each acetabulum.

**Figure 7.**
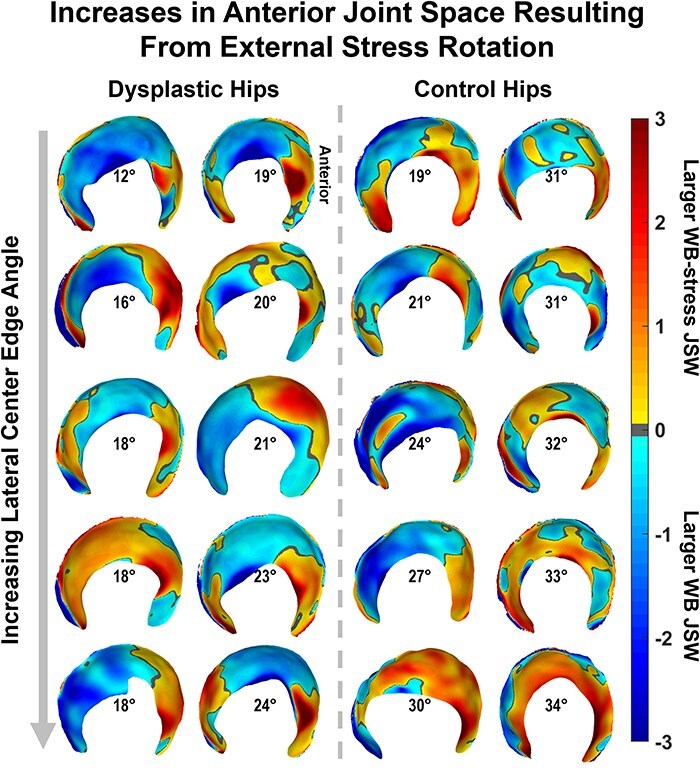
JSW differences from the unstressed to the stressed orientation in the WBCT scanner produced noticeable anterior increases in both cohorts that were not apparent from statistical testing, with both groups (dysplastic: left; control: right) ordered by increasing lateral coverage (LCEA) shown in the center of each acetabulum.

There were minimal differences in average regional or average whole-hip JSW (Table 2) between the WB and NWB scans for dysplastic hips. However, when considering each hip individually, more than half of the dysplastic hips had smaller anterior and medial JSW in NWB than in WB stance (Fig. 6). Dysplastic hips also had smaller whole-hip JSW (*P *= .065) in the WB-stress stance compared to the WB stance, indicating overall compression of the joint that was unobserved in control hips (*P *= .710). Local JSW changes in the dysplastic hip from WB-stress included increased anterior (lateral: *P *= .020; medial: *P *= .055) and decreased superior (lateral: *P *= .090; medial: *P *= .027) JSW (Table 2). Control hips exhibited less consistent changes in local JSW from stress (Table 2), including widening of the anterior joint space (Fig. 7).

## Discussion

WBCT is a beneficial tool that employs lower radiation dosage in faster acquisition times compared to standard imaging studies and conventional CT [28–31, 32]. There is currently minimal research on its application in the hip [27], but it has been established as an effective clinical tool to evaluate foot, ankle, and knee alignment in a standing, loaded position [24, 25, 28, 37–40]. The aim of this study was to determine if femoral–acetabular displacement can be measured on WBCT. Given the image quality (influenced by current WBCT technology and motion artifact), 2D measurements had variably low interobserver reliability (0.002–0.725). The 3D computational assessment of femoral head translation and JSW was automated and thereby provided a more objective indicator. Contrary to our hypothesis, 3D femoral head center translation resulting from external rotation stress tended to be larger in nondysplastic control hips than in dysplastic hips, and overall JSW was larger in the control hips than in the dysplastic hips in both unstressed and stressed standing WBCT conditions. The WB-stress scans showed regionally greater anterior and smaller superior JSW compared to the WB scans in dysplastic hips; however, there was not a reliably strong trend in any regional JSW measurement that would indicate this external rotation stress test elicited substantial anterior joint instability.

While objective measurement and classification of hip instability have been explored by various techniques, this study is the first to utilize a weight-bearing cone-beam CT scanner to investigate femoral–acetabular displacement in orientations that may cause hip instability. Previous studies evaluating hip instability with clinical imaging have measured 2D anterior coverage with pelvic tilt on standing AP radiographs [41], subluxation with external rotation on dynamic MRI [42], and joint translation on standing AP and bilateral 45 Dunn radiographs [43]. Similar to our results, those previous studies have a wide range of inter/intra-rater reliability from excellent [43] to moderate/poor [41, 42]. The objectivity of quantitative 3D measures of instability is therefore appealing, as is the ability to assess the whole joint, rather than a single plane [44–48]. The specific 3D measurements used in this work included a well-established JSW technique [44–47, 49–52] and femoral head translation [53–57]. This combination of 3D measures enabled identification of more position- and cohort-dependent differences in femoral–acetabular displacement.

WBCT was used in this study to directly obtain functional bone orientations with clinically relevant imaging, but we recognize that other, less clinically common pathways could have been utilized. For example, registering NWB CT-derived geometry to standing AP radiographs can facilitate measurement of FHC translation, as was done in a recent study that found FHC translation from supine to standing in borderline (18° ≤ LCEA < 25°) dysplastic hips was on average 2.2 mm [54]. Another approach is the use of biplane-fluoroscopy, which had been utilized to measure 3D FHC translation, finding translations of 0.9–1.3 mm in asymptomatic control hips associated with the transition from a static standing position to a weight-bearing apprehension position [55]. While these approaches quantify 3D functional orientations, their clinical applicability is limited. Our novel use of WBCT imaging revealed femoral head translations of 0.5 mm in dysplastic hips (NWB to WB) and 1.3 mm in control hips (WB to WB-stress), highlighting the ease of capturing functional orientations with this clinically relevant method and its future potential to enhance clinical decision-making in the future.

There are several limitations to this first application of standing WBCT to evaluate femoral–acetabular displacement. The first is the small sample size (*n* = 10) for both groups and the relatively mild deformity in the dysplasia group (LCEA range: 12–24°), making it impossible to determine what relationship may exist between abnormal femoral head movement and the severity of dysplastic deformity. Further, we excluded patients with established instability conditions, such as Ehlers-Danlos syndrome, which reduced the magnitudes of the femoral head movement found in this work. In future studies, it will be critical to collect fully equivalent imaging studies for cohorts with a wider range of dysplasia severity or soft tissue conditions to determine if WBCT technology can add to or replace clinical CT scans for evaluating femoral head translations indicative of hip instability. Another limitation of this work was the low inter-observer reliability of most 2D CT measurements. While previous studies of distal tibiofibular syndesmosis congruity report inter-rater reliability values from 0.661 to 0.964 for WBCT [39] and from 0.466 to 0.9 for NWB CT [58], agreement in 2D measurements in this work ranged from 0 to 0.5. This may reflect the current image quality of WBCT compared to standard CT. While images generated by the WBCT scanner were acceptable for many clinical purposes, the reconstruction algorithms for the scanner would benefit from optimization to best visualize the hip joint. In particular, increased image contrast and decreased noise would improve image quality, thereby hopefully increasing measurement reliability. Furthermore, the patient stabilizer on the scanner could not be adjusted to support patients during the unique external stress scan pose, which introduced greater levels of motion artifact. Future work would benefit from an improved emphasis on patient stabilization to maximize the quality of images available for analysis. Increased patient stabilization would also enable testing patients in other positions that elicit instability beyond the clinically relevant external rotation explored within this body of work. However, the limitations in patient positioning imposed by the scanner did not permit testing deep hip flexion (e.g. posterior instability). Lastly, detecting JSW through ray-cast projection and femoral head translation through sphere-fitting of reconstructed bony geometry is limited by the resolution of the CT scans; higher scan resolution and reduced slice thicknesses may improve the ability to capture more subtle changes in hip motion.

In conclusion, WBCT offers an alternative to standard supine clinical CT that allows for 3D imaging of the hip in a functionally loaded position. Two-dimensional assessment of femoral head center location in the acetabulum was unreliable and did not identify strong differences in femoral head movement between dysplastic and control hips or between standing and externally rotated positions in either hip type. Implementation of 3D measurements found differences in femoral-acetabular displacement that were patient-specific and independent of acetabular coverage. Further development of the WBCT technology, as well as its application in large population studies, holds potential to provide quantitative assessment of hip instability and aid in surgical decision-making for cases of mild or borderline hip dysplasia.

## Data Availability

The authors confirm that all data generated or analyzed during this study are included in this published article. Raw data that supports the findings of this study are available, upon reasonable request, from the corresponding author.
